# Utilizing Network Pharmacology and Molecular Docking Integrated Surface Plasmon Resonance Technology to Investigate the Potential Targets and Mechanisms of *Tripterygium wilfordii* against Pulmonary Artery Hypertension

**DOI:** 10.1155/2022/9862733

**Published:** 2022-04-30

**Authors:** Shifa Wang, Yunjing Liu, Qingguo Wang, Xiufeng Xu, Tao Huang, Peikang Dong, Lide Wang, Bufan Cao, Qiuhong Jiao, Xiaodong Sun, Jingtian Li, Tao Wang

**Affiliations:** ^1^Department of Cardiology, Affiliated Hospital of Weifang Medical University, Weifang 261031, Shandong, China; ^2^Department of Neurology, Affiliated Hospital of Weifang Medical University, Weifang 261031, Shandong, China; ^3^Department of Endocrinology, Affiliated Hospital of Weifang Medical University, Weifang 261031, Shandong, China

## Abstract

**Background:**

Pulmonary artery hypertension (PAH) is a rare, life-limiting cardiopulmonary disorder characterized by the progressive and remodeling of pulmonary vasculature. Although the development of the technology brings us many approaches for the treatment of PAH, the effect of treatment is unsatisfactory. *Tripterygium wilfordii* (TW), as a traditional Chinese medicine (TCM), has been widely used in anti-inflammation, anticancer, and other fields. However, the potential of TW in treating PAH is currently unclear.

**Methods:**

Active ingredients and their corresponding genes were harvested from the Traditional Chinese Medicine Database and Analysis Platform (TCMSP), CTD, and STITCH. Meanwhile, genes associated with PAH were adopted from OMIM and GeneCards databases. Through Gene Ontology (GO) and Kyoto Encyclopaedia of Genes and Genomes (KEGG) pathway enrichment analyses, potential targeting KEGG pathways and functions were further collected. Then, STRING was used to generate the protein-protein interaction (PPI) network. The “ingredients-targets-pathway” network was built by Cystoscope. Finally, the binding between active ingredients of TW and corresponding targets of PAH was identified *via* molecular docking technology and surface plasmon resonance (SPR) experiments.

**Results:**

The network pharmacology analysis revealed 36 active ingredients in TW and 150 potential targets related to the treatment of PAH with TW. Moreover, GO enrichment analysis showed that the key function in molecular function (MF) was related to enzyme binding, the key function in biological process (BP) was related to cellular response to organic substance, and the key function in cellular component (CC) was related to KEGG enrichment analysis and found that it was closely related to the IL-17 signaling pathway, TNF signaling pathway, Toll-like receptor signaling pathway, and apoptosis. At last, molecular docking results revealed that the main active ingredients of TW had a strong binding ability with the PAH target protein. In addition, the SPR experiment revealed that kaempferol was combined with the CASP3 protein rather than PARP1, while triptolide was combined with PARP1 rather than the CASP3 protein.

**Conclusion:**

TW may have therapeutic effects on PAH through multitargets and multimethods, which provide a scientific basis for further elaborating the mechanism of *Tripterygium wilfordii* in the treatment of PAH.

## 1. Introduction

Pulmonary artery hypertension (PAH) is a common chronic disease in clinical practice, which is the main category of pH classification in the Second World Symposium [1, 2]. Although there are many influencing factors of pulmonary artery hypertension (PAH), such as age, living habits, race, and gender, most patients diagnosed with PAH are young women [3]. Without correct diagnosis and appropriate treatment, it may aggravate and cause many serious consequences, such as right ventricular (RV) failure and ultimately death [4]. The pathophysiological mechanisms of PAH are complex and may be related to genetic factors, immune/inflammatory responses, and environmental factors (e.g., dasatinib or methamphetamine) [5]. At present, the studies of PAH are mainly concentrated in the following aspects, including the mechanism of cancer cell dysfunction, cellular metabolic disorders, and abnormal proliferation [6]. PAH has been increasingly received scholars' study in the last few years. Though researches have focused on the pathogenesis of PAH, its accurate mechanism has not been fully clarified, and current pharmacological treatments still have many shortcomings. Therefore, it is necessary to study the mechanism of traditional Chinese herbal medicine for PAH treatment.

*Tripterygium wilfordii*, a traditional Chinese herbal medicine, has been widely used in various diseases, such as rheumatoid arthritis (RA), systemic lupus erythematosus (SLE), and psoriasis [7]. Its components mainly contain alkaloids, diterpenoids, and polysaccharides, which have played an important role in many fields, such as anti-inflammation, oxidation, and nerve protection.

Network pharmacology, a novel concept proposed by British pharmacologist Hopkins, systematically revealed the mechanisms by which drugs act on a disease [8]. By constructing and analyzing the relationship model among drugs, diseases, and related targets of action, a network of the three was formed to systematically reveal the mechanism of drug action on a disease. For the analysis of multicomponent, multitarget, and multimechanism drugs of traditional Chinese medicine, this method was especially suitable [9]. At present, network pharmacology involves the following steps in clinical application: screening active pharmaceutical ingredients and targets, collecting targets for diseases, predicting protein-related targets, and finding related KEGG pathways in which drugs act. Its mechanism of action could be better understood at the molecular level by analyzing drug-target interactions [10, 11]. This method could accurately distinguish potential drug-target interactions, providing a theoretical basis for clinical studies. Therefore, network pharmacology provided concise and ample evidence in guiding clinical practice.

**Figure 1:**
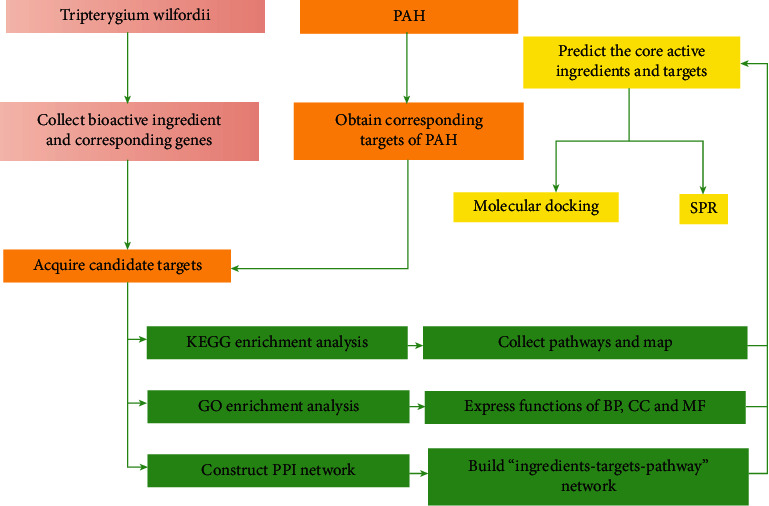
A framework of analysis procedures to identify the potential targets and mechanisms of TW against PAH.

In our study, the main active substances, active targets, and signaling pathways of Tripterygium wilfordii were investigated by the network pharmacology method, molecular docking, and SPR (surface plasmon resonance) experiment verification methods to provide new ideas for the treatment of PAH by *Tripterygium w ilfordii* (Figure 1).

## 2. Materials and Methods

### 2.1. Collection of Bioactive Ingredients of TW

The bioactive ingredients of TW are filtered from Traditional Chinese Medicine Systems Pharmacology (TCMSP) (https://tcmspw.com/tcmsp.php), which is an analysis platform that represents ideal information convergence of pharmacochemistry, absorption, distribution, metabolism, and excretion (ADME) properties, drug-likeness, drug targets, associated diseases, and interaction networks [12]. The species type was set to “*Homo sapiens.*” The bioactive components were obtained by the metrics of oral bioavailability (OB) > 30%, drug-likeness (DL) > 0.18, and Caco-2 > 0.

### 2.2. Screening Corresponding Genes of TW

CTD (https://ctdbase.org/) and STITCH (https://stitch.embl.de/) are widely used to collect the corresponding genes of TW. CTD is a public database, which provides relations of gene products, diseases, and chemical and environmental exposures [13]. STITCH is an analysis system that predicts the target proteins/genes of plant components [14]. Organisms equal to *Homo sapiens* were limited. We used the screened genes to conduct further analysis.

### 2.3. Obtaining Corresponding Targets of PAH

Potential targets of PAH were obtained from GeneCards (https://www.genecards.org/) and the OMIM database (https://www.omim.org/). OMIM is a sophisticated, authoritative, and timely research resource that provides an elaborate description of human genes, phenotypes, and their relationships [15]. GeneCards is a one-stop shop to hunt for human gene annotations, which is the root of at least 120 sources and offers compositive information for every human gene [16]. The species type was limited to “*Homo sapiens*”. The keyword “pulmonary artery hypertension” was inputted into the search for the target genes of PAH from the two databases.

### 2.4. Acquisition of Candidate Targets

The Venn diagram, which consisted of the obtained genes, was drawn by the R Venn package (version 1.9). Overlapping genes between TW and PAH were identified as candidate genes.

### 2.5. Gene Ontology (GO) and Kyoto Encyclopaedia of Genes and Genomes (KEGG) Enrichment Analysis

The Gene Ontology (GO) category database and the KEGG database are used for functional annotation of candidate genes. Enrichment analysis of GO categories, composed of cellular components (CC), biological process (BP), and molecular function (MF), was performed by the R cluster Profiler (v3.14.3) package, and KEGG enrichment analysis of pathways was tested upon hypergeometric distribution by R “phyper” function. Those GO categories with a false discovery rate (FDR) <0.05 were considered as significantly enriched. However, KEGG pathways with a *p* value <0.05 were regarded as enriched. Only those GO categories or KEGG pathways contained ≥5 DEGs were kept for further analysis.

### 2.6. Construction of Protein-Protein Interaction (PPI) Network

The hub proteins were inputted into STRING (https://string-db.org) with the species limited to *Homo sapiens* and the highest confidence >0.9. The STRING database is to collect and merge bioinformation by integrating known and predicted the relations of protein-protein for organisms [17]. Molecular Complex Detection (MCODE) could adopt the crucial cluster of the PPI network. These clusters were provided by enrichment analysis. The PPI network was visualized by Cytoscape (version 3.6.1), which was an opened source software program for the visualization of interaction between elements [18].

### 2.7. Construction of the “Ingredients-Targets-Pathway” Network

The “Ingredients-targets-pathway” network was visualized by Cytoscape software. The node's attributes of this network like degree, betweenness, and closeness centrality were calculated by the Network Analysis tool. Betweenness, one of the structural measures in the network, is used to compare the importance of target nodes [19]. Closeness centrality is a well-known structural measure, and its functions for disease gene prediction on undirected biological networks have been frequently reported [20].

### 2.8. Molecular Docking

The crystal structures of CASP3 (PBD ID: ICP3) and PARP1 (PBD ID: IUKO) were obtained from the RCSB PDB database (https://www.rcsb.org/). The chemical structures of kaempferol and triptolide were retrieved from the PubChem database (https://pubchem.ncbi.nlm.nih.gov/). The molecular docking studies between proteins and compounds were based on these obtained structures and carried out with the AutoDock Package (version 4.2). The AutoDock suite provides a comprehensive toolset for computational ligand docking and drug design and development, including empirical free energy force fields, docking engines, methods for site prediction, and interactive tools for visualization and analysis [21]. Potential binding sites within the crystal structures were predicted based on grid energy calculation *via* the AutoGrid program. Conformation searching and energy evaluation were completed with the AutoDock program. AMBER Tools (version 14) was performed to dock the active ingredients with protein receptor molecules, and AMBER Tools MM/PBSA was used to visualize the affinities of active ingredients and targets. Briefly, the optimal complex was placed in a cubic water box with a minimum distance of 10 Å between protein surface and the box edges. Proper sodium was added to achieve physiological salt conditions with overall neutrality. Energy minimization was performed under the force field. The constraint of all bonds involving hydrogen atoms was achieved with the SHAKE method. Average structures were extracted for interaction mode analysis.

### 2.9. SPR Experiment

The Sensor Chip NTA (SEN-AU-100-10-NTA, SNC1006, Nicoya) should be prepared before the experiment, which was performed with an OpenSPRTM surface plasmon resonance instrument (Nicoya). The Casp3 (10050-H08E, SinoBiological) and PARP1 (11040-H08B, SinoBiological) proteins were diluted to 30 *μ*g/mL in immobilization buffer (1× PBS, pH = 7.4). Imidazole and NiCl_2_ solutions were injected into the activated chip to complete the surface functionalization of the chip. Then, Casp3 and PARP1 proteins were injected at a flow rate of 20 *μ*L/min, and the capture levels were respectively reached at 1000 RU and 1200 RU. Micromolecules were diluted with Running Buffer (1 × PBS with 1% DMSO, pH = 7.4) and injected into the flow cell of the channel at a flow rate of 20 *μ*L/min for an association of 240 s, followed by 300 s dissociation. Both the association and dissociation processes were handled with the Running Buffer. Repeat 5 cycles of analyte according to analyte concentrations in ascending order. After each cycle of interaction analysis, the sensor chip surface should be regenerated completely with PBS as the injection buffer at a flow rate of 100 *μ*L/min for 30 s to remove the ligand and any bound analyte. The analysis software used in this experiment was TraceDrawer (Ridgeview Instruments ab Sweden) and was analyzed by the one-to-one analysis model.

## 3. Results

### 3.1. Collection of Bioactive Ingredients and Screening for Corresponding Genes of TW

**Figure 2:**
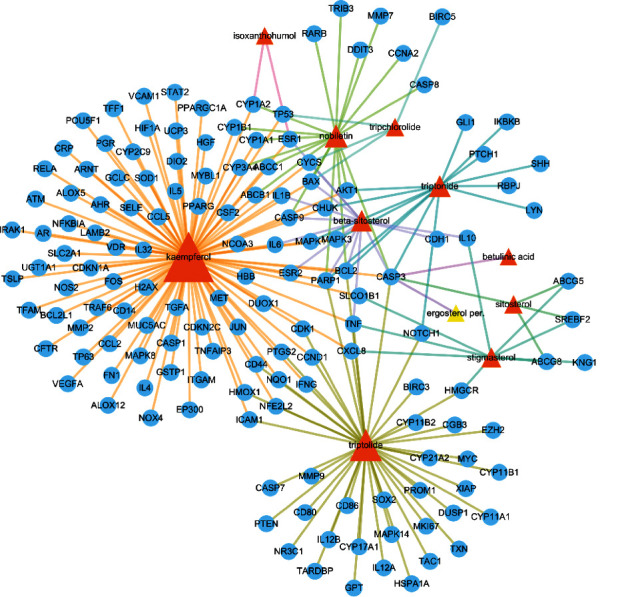
Network model of bioactive ingredient-target. The orange nodes represent the bioactive ingredients of TW. The blue nodes indicate the corresponding genes of TW.

The TCMSP database was used to collect the active ingredients in TW. It is shown in Table 1. We found that 36 active ingredients were identified by the limitations to OB > 30%, DL > 0.18, and Caco > 0, mainly including kaempferol, celaxanthin, celafurine, stigmasterol, nobiletin, and triptolide. Next, we searched for 207 corresponding genes via CTD and STITCH databases. After 25 ingredients without target were removed, the “ingredient-target” network (Figure 2) was constructed by Cytoscape software. The “ingredient-target” network contained 158 nodes (including 11 ingredients and 147 targets) and 208 edges. The red triangle and blue circle represented the ingredients and targets, respectively.

### 3.2. Obtaining Corresponding Targets of PAH and Acquisition of Candidate Targets

**Figure 3:**
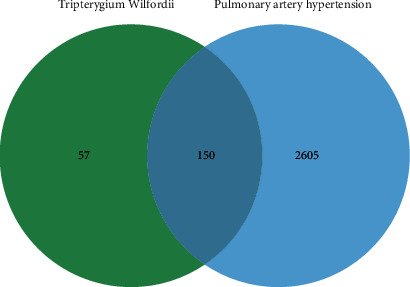
The Venn diagram of the overlapping targets of TW and PAH. The green circle was described as the genes of TW. The blue circle was represented as the genes of PAH. The common part of the circle was regarded as the overlapping genes.

In the present study, 2755 genes of PAH were screened out by GeneCards and the OMIM database. The R Venn package was used to draw the Venn diagram after filtering the obtained genes of TW and PAH. A total of 150 overlapping genes were filtered as candidate targets. Venn diagram data are shown in Figure 3.

### 3.3. Gene Ontology (GO) and Kyoto Encyclopaedia of Genes and Genomes (KEGG) Enrichment Analysis

**Figure 4:**
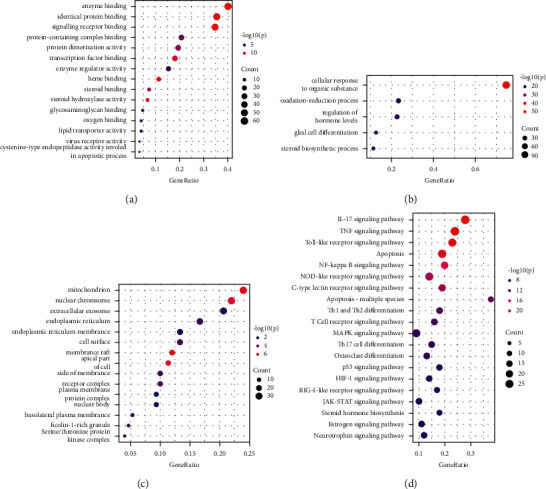
The GO and KEGG pathway enrichment analyses of potential targets of active ingredients of TW against PAH. (a) The top 15 significant enriched terms in molecular function (MF). (b) The top 5 significant enriched terms in biological process (BP). (c) The top 15 significant enriched terms in cellular component (CC). (d) The top 20 significant enriched terms in KEGG analysis.

**Figure 5:**
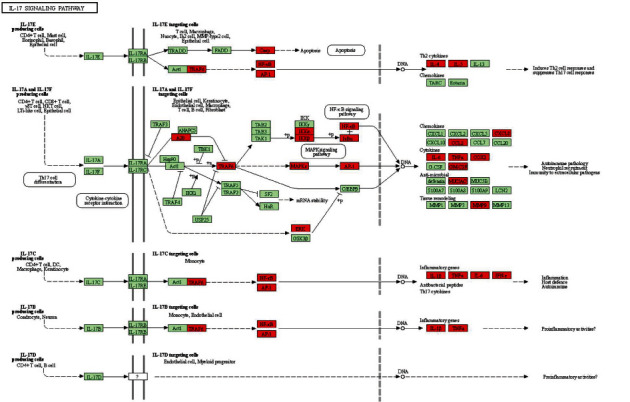
Schematic drawing to illustrate the significant signaling pathways of TW against PAH. (a) Schematic drawing of IL-17 signaling pathway. (b) Schematic drawing of TNF signaling pathway. (c) Schematic drawing of Toll-like receptor signaling pathway. (d) Schematic drawing of apoptosis.

GO enrichment was obtained in our study, which expressed the functions of collected genes in Biological Process (BP) (Figure 4(a)), cytological component (CC) (Figure 4(b)), and molecular function (MF) (Figure 4(c)). The key function in BP was related to cellular response to organic substances. The key function in CC was related to mitochondrion. The key function in MF was related to enzyme binding. We could search the signaling pathway from KEGG enrichment analysis (Figure 4(d)), mainly including the IL-17 signaling pathway, TNF signaling pathway, Toll-like receptor signaling pathway, and apoptosis. In addition, we built a target-pathway network that demonstrated the important role of core targets in the signaling pathways (Figure 5).

### 3.4. Protein-Protein Interaction (PPI) Network Construction

**Figure 6:**
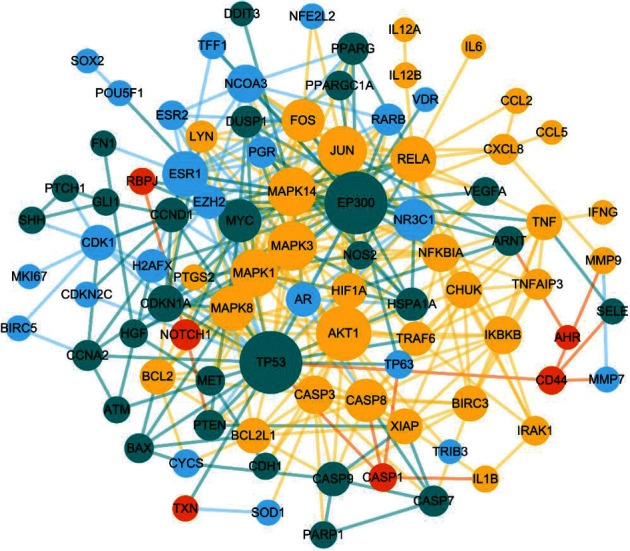
The protein-protein interaction (PPI) network of TW and PAH. The nodes indicated potential targets. The edges indicated the relationships between the two targets.

**Figure 7:**
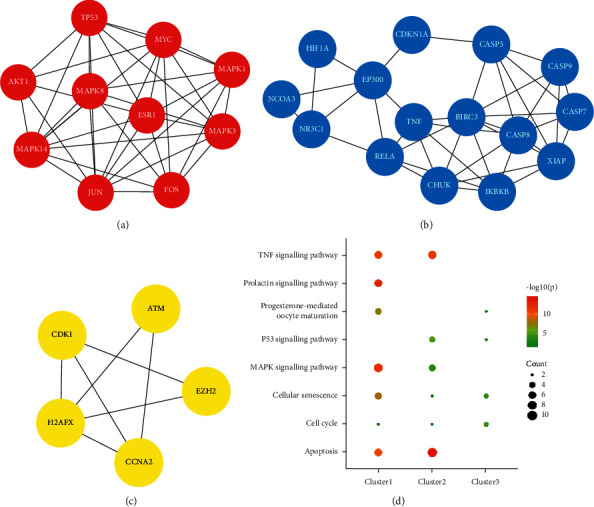
The networks of the core targets of clusters in this study. (a) The red cluster represented MCODE1. (b) The blue cluster represented MCODE2. (c) The yellow cluster represented MCODE3. (d). The KEGG enrichment analysis of clusters. The top 7 significant KEGG pathways of cluster 1 in the KEGG enrichment analysis. The top 6 significant KEGG pathways of cluster 2 in the KEGG enrichment analysis. The top 4 significant KEGG pathways of cluster 1 in the KEGG enrichment analysis.

The 150 obtained targets were imported into the STRING database to filter protein interrelationships, which were visualized into the protein-protein interaction (PPI) network by Cytoscape software (Figure 6). The network included a total of 90 nodes, which might play an important role in the treatment of PAH with TW. Under the criteria of *p* value, the three clusters with the best scores in the PPI network were selected to describe their biological functions (Figure 7). In summary, the functions mainly involved many important physiological and pathological processes, such as endocrine resistance, apoptosis, and cell cycle (Table 2).

### 3.5. Construction of the “Ingredients-Targets-Pathway” Network

**Figure 8:**
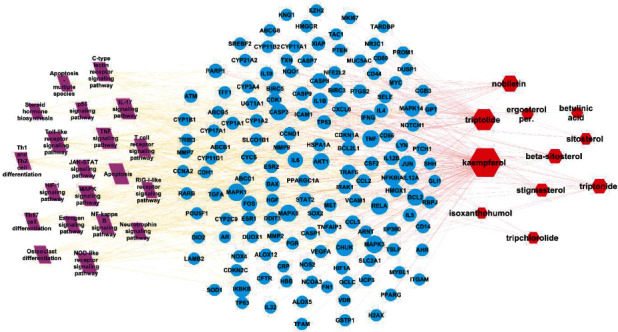
The ingredient-target-pathway network. The red nodes indicated the active ingredients. The blue nodes indicated the potential targets in TW against PAH. The purple nodes indicated the KEGG pathways in TW in the treatment of PAH.

Cytoscape software was used to construct the active component-target-PAH pathway network model of *Tripterygium wilfordii* (Figure 8). The topology of each node in the network was evaluated by degree and betweenness degree. We found that the network had 178 nodes (including 11 ingredients, 147 targets, and 20 KEGG pathways) and 577 relationship pairs in the journey. It could be seen that TW played a role in mediating anti-inflammatory, hypoxic perception, apoptosis, and response through multiple pathways and multiple targets, thereby treating PAH on the overall level. The highest degree of components was kaempferol. It had the highest degree and the closeness centrality. It was predicted that kaempferol is the core component for PAH intervention, followed by triptolide, nobiletin, triptonide, beta-sitosterol, and stigmasterol (Table 3). CASP3 had the highest degree, betweenness centrality, and closeness centrality. It was predicted that CASP3 was a core target for PAH intervention, and PARP1, BCL2, Bax, and TNF were also relatively important targets (Table 4).

### 3.6. Molecular Docking and SPR Experiment

**Figure 9:**
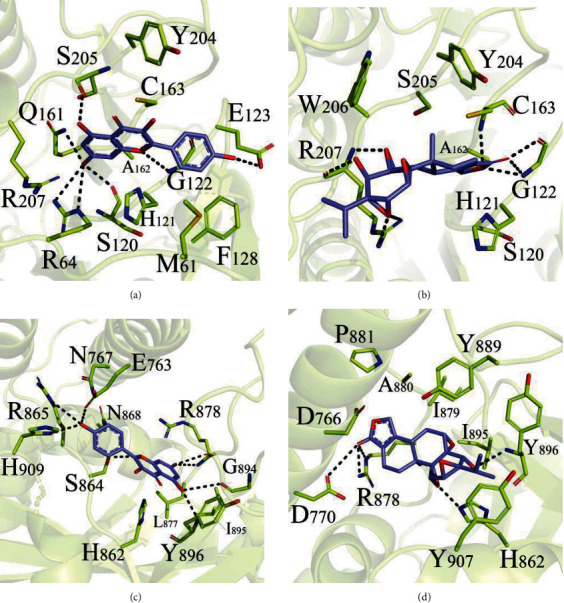
The schematic diagram of molecular docking. (a) Kaempferol and ICP3-CASP3; (b) triptolide and ICP3-CASP3; (c) kaempferol and IUKO-PARP1; (d) triptolide and IUKO-PARP1.

Auto Dock was used to predict the binding energy of the two core target proteins (CASP3 and PARP1) corresponding to the top 2 active ingredients (kaempferol and triptolide) of *Tripterygium wilfordii* (Figure 9). The more stable the conformation, the lower the binding energy. A docking score <−5.0 kcal/mol indicated that the ingredients had a good binding activity to the protein targets, while a docking score <−7.0 kcal/mol indicated a strong binding activity [22]. The results of molecular docking showed that the binding scores were less than −7.0 kcal/mol, indicating the strong binding activity between the target proteins (CASP3 and PARP1) and the components (kaempferol and triptolide). Kaempferol had the highest affinity with CASP3 (Table 5). The experiment indicated that kaempferol was combined with CASP3 protein (−8.46 kcal/mol) rather than PARP1 (−7.52 kcal/mol), while triptolide was combined with PARP1 (−8.35 kcal/mol) rather than CASP3 protein (−7.67 kcal/mol). The active compound of *Tripterygium wilfordii* was mainly bound to the target through hydrogen bonding, hydrophobic interaction, and -OH phenol interaction, which provided a strong binding force for the connections of the active compounds and the target proteins.

### 3.7. SPR Experiment

**Figure 10:**
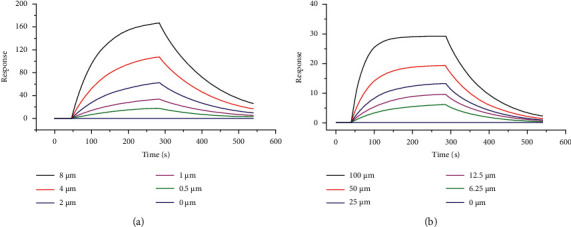
The results of SPR experiment. (a) Kaempferol combined with CASP3 protein. (b) Triptolide combined with PARP1 protein.

A surface plasmon resonance (SPR) experiment was a kind of ligand with membrane protein interaction technology that could be used to verify the drug's main molecular targets. The experiment was unlabeled and was able to measure the real-time quantitative binding affinity and kinetics of the interaction of membrane proteins with ligand molecules using a relatively small amount of material [23]. The related constants of the SPR experiment are obtained in Table 6. The SPR experiment revealed that Casp3 protein, captured on Sensor Chip NTA, could bind kaempferol with an affinity constant of 7.65 µM as determined in the SPR (Figure 10(a)). PARP1 protein, captured on Sensor Chip NTA, could bind with an affinity constant of 41.8 *μ*M as determined in the SPR (Figure 10(b)).

## 4. Discussion

As a method for the analysis of multicomponent, multitarget, and multimechanism herbal medicines, network pharmacology has been increasingly used in clinical practice. It has systematically revealed the mechanism by which herbal medicines act on the disease [8]. And it constructed and analyzed a model of the relationship among drugs, diseases, and related targets of action and formed a network among them. In this present study, a total of 36 active ingredients and 270 predicted targets of *Tripterygium wilfordii* were obtained, which together constituted a network diagram of the disease-active ingredient-predicted targets. The pathogenesis and development of pulmonary artery hypertension is a complex process involving various risk factors in multiple systems. Similarly, herbal medicines also play an important role in diseases by acting on different active components and targets. This study attempted to better explain the mechanism of action of *Tripterygium wilfordii* on pulmonary artery hypertension using network pharmacology and further verified its results by molecular docking, and the SPR experiment was further used to validate the binding relationship between ingredients and targets of this herbal medicine.

As a common pulmonary vascular disease in clinical practice, pulmonary artery hypertension was characterized by a progressive increase in pulmonary vascular resistance, which could lead to various clinical complications, ultimately lead to right heart failure or even death [1, 4]. Its mortality and morbidity are high. Therefore, the current attention to pulmonary artery hypertension has gradually increased. At present, the treatment of pulmonary artery hypertension was mainly based on the treatment of primary diseases. In addition, there have been many drugs that can treat this disease in clinical practice, such as sildenafil and beraprost sodium. However, the mechanism of *Tripterygium wilfordii* in pulmonary artery hypertension is not clear.

In this study, we concluded by network pharmacology and molecular docking that the compounds with the highest comprehensive score of *Tripterygium wilfordii* was kaempferol, followed by triptolide, sichenesin, and *Tripterygium wilfordii* lactone. Kaempferol has many pharmacological activities such as antioxidant damage, immune regulation, and estrogen-like effect, and has preventive and therapeutic effects on RA, diabetes, osteoporosis, and cardiovascular diseases. Kaempferol could be used in the treatment of cancer. Relevant studies have found that kaempferol can affect autophagy in cancer cells, thereby exerting antitumor activity [24]. Many studies have found that triptolide has a wide range of antiproliferative effects. Triptolide had the effect of reducing inflammatory cell infiltration and reducing the number of B lymphocytes and plasma cells [25]. Relevant experimental studies have found that nobiletin shows a significant effect by inhibiting the proteins and their mRNAs of IL-1a, IL-1*β*, IL-6, and TNF-*α* in mice macrophages. In addition, it has been found that nobiletin acts by increasing antioxidant activity and characteristics [26].

The Gene Ontology project classifies functions based on three aspects: biological processes, cellular components, and molecular functions. It involves cellular response to organismal substances, membrane rafts, signaling receptor binding, etc. The KEGG enrichment analysis results showed that the targets were regulated by 243 KEGG pathways. It was related to multiple biological processes and signaling pathways associated with *Tripterygium wilfordii* treating PAH, including the IL-17 signaling pathway, TNF signaling pathway, Toll-like receptor signaling pathway, and apoptosis. Studies have found that the IL-17 function of interleukin (IL)-17, as one member of the new family members of inflammatory cytokines, is much more subtle than that it simply triggers inflammation. Increasing evidence suggests that IL-17 has important environmental and tissue-dependent roles in maintaining health in response to injury, physiological stress, and infection [27, 28]. Relevant studies have shown that the increase in TNF-*α* can cause the abnormality of nitric oxide lyase (NOS) in vascular endothelial cells, reduce the synthesis of prostaglandin (PG) by pulmonary artery smooth muscle cells, and eventually lead to impaired vascular endothelial function and pulmonary vasoconstriction. Studies have shown that kaempferol might be involved in NF-*κ*B signaling pathway to inhibit the expression of the major proinflammatory cytokines, including IL-17A and TNF-*α*, and decrease the percentage of IL17A^+^CD4^+^ T cells in the imiquimod (IMQ)-induced psoriatic mouse model [29]. In addition, kaempferol, genistein, and glycitein disrupt interleukin (IL)-6R, toll-like receptor (TLR)-4 and their respective ligands interaction to hinder the expression of HIF-1, which is associated with the phosphoinositide 3-kinases (PI3K)/Akt signaling pathway [30]. Relevant studies have found that TLRs are transmembrane pattern recognition receptors that play an important role in the detection and defense of microbial pathogens in natural immunity [31]. It is considered that *Tripterygium wilfordii* treatment of PAH may be related to these KEGG pathways, and further validation is required.

To further verify the mechanism of action, we used molecular docking to predict and analyze the binding energy of the active components of *Tripterygium wilfordii* and their corresponding active targets. kaempferol and CASP3 obtained the highest docking total score, which was higher than the docking total score for the binding of triptolide and PARP1. For further validation, we performed SPR experiments. It showed that kaempferol and triptolide were selected as extracellular validation molecular models to perform small-molecule and macromolecule interaction experiments with CASP3 and PARP1, respectively. According to the results, it can be speculated that kaempferol and triptolide can bind to CASP3 and PARP1 and inactivate them so as to prevent other proteins from binding to cell surface receptors. In summary, these KEGG pathways acted synergistically in the treatment of PAH, providing a strong basis for multitarget therapy. In addition, molecular docking has been widely used in the prediction of clinical targets. Molecular docking results showed that kaempferol and triptolide had a high score when docking with CASP3 and PARP1, and this result suggested that further attempts could be made to develop their leading compounds, which was consistent with the results of network pharmacology analysis.

## 5. Conclusion

TW may have therapeutic effects on PAH through multitargets and multimethods, which provided a scientific basis for further elaborating the mechanism of *Tripterygium wilfordii* in the treatment of PAH.

### 5.1. Future Perspective

TW, as a traditional Chinese medicine, has been increasingly valued in the treatment of various diseases, and many mechanistic studies on TW and diseases are under way. To our knowledge, there is no study performed on TW and PAH mechanisms. In this study, the mechanisms between TW and PAH were investigated by network pharmacology, molecular docking, and SPR trials. However, the specific pathways in the treatment of PAH are unclear. The efficacy of TW in treating PAH through multiple pathways and multiple targets also needs further experimental verification. It may provide new perspectives on the treatment of PAH and contribute to the development of Chinese herbal medicine in the treatment of diseases.

## Figures and Tables

**Table 1:** The main components of *Tripterygium wilfordii*.

Molecule ID	Compound name	OB	DL	Hdon	Hacc
MOL000296	Hederagenin	36.91	0.75	1	1
MOL003182	(+)-Medioresinol di-O-beta-D-glucopyranoside_qt	60.69	0.62	2	7
MOL003184	81827-74-9	45.42	0.53	1	4
MOL003185	(1R,4aR,10aS)-5-Hydroxy-1-(hydroxymethyl)-7-isopropyl-8-methoxy-1,4a-dimethyl-4,9,10,10a-tetrahydro-3H-phenanthren-2-one	48.84	0.38	2	4
MOL003187	Triptolide	51.29	0.68	1	6
MOL003188	Tripchlorolide	78.72	0.72	2	6
MOL003189	Wilforlide A	35.66	0.72	2	4
MOL003192	Triptonide	67.66	0.7	0	6
MOL003196	Tryptophenolide	48.5	0.44	1	3
MOL003199	5,8-Dihydroxy-7-(4-hydroxy-5-methyl-coumarin-3)-coumarin	61.85	0.54	3	7
MOL003206	Canin	77.41	0.33	1	5
MOL003208	Celafurine	72.94	0.44	2	6
MOL003209	Celallocinnine	83.47	0.59	2	5
MOL003211	Celaxanthin	47.37	0.58	1	1
MOL003217	Isoxanthohumol	56.81	0.39	2	5
MOL003225	Hypodiolide A	76.13	0.49	1	3
MOL003229	B Triptinin B	34.73	0.32	2	3
MOL003231	Triptoditerpenic acid B	40.02	0.36	1	3
MOL003232	Triptofordin B1	39.55	0.84	1	6
MOL003244	Triptonide	68.45	0.68	0	6
MOL003245	Triptonoditerpenic acid	42.56	0.39	2	4
MOL003248	Triptonoterpene	48.57	0.28	1	2
MOL003266	21-Hydroxy-30-norhopan-22-one	34.11	0.77	1	2
MOL003278	Salaspermic acid	32.19	0.63	2	4
MOL003280	TRIPTONOLIDE	49.51	0.49	1	4
MOL000358	Beta-sitosterol	36.91	0.75	1	1
MOL000211	Mairin	55.38	0.78	2	3
MOL000422	Kaempferol	41.88	0.24	4	6
MOL000449	Stigmasterol	43.83	0.76	1	1
MOL002058	40957-99-1	57.2	0.62	2	7
MOL004443	Zhebeiresinol	58.72	0.19	1	6
MOL005828	Nobiletin	61.67	0.52	0	8
MOL007415	[(2S)-2-[[(2S)-2-(Benzoylamino)-3-phenylpropanoyl]amino]-3-phenylpropyl] acetate	58.02	0.52	2	6
MOL007535	(5S,8S,9S,10R,13R,14S,17R)-17-[(1R,4R)-4-Ethyl-1,5-dimethylhexyl]-10,13-dimethyl-2,4,5,7,8,9,11,12,14,15,16,17-dodecahydro-1H-cyclopenta[a]phenanthrene-3,6-dione	33.12	0.79	0	2
MOL009386	3,3′-Bis-(3,4-dihydro-4-hydroxy-6-methoxy)-2H-1-benzopyran	52.11	0.54	2	6
MOL011169	Peroxyergosterol	44.39	0.82	1	3

**Table 2:** MCODE function description.

MCODE	Path ID	Function description	Log10(P)
MCODE1	hsa01522	Endocrine resistance	−16.4
	hsa05161	Hepatitis B	−14.4
	hsa05210	Colorectal cancer	−14.3

MCODE2	hsa04210	Apoptosis	−14.4
	hsa05200	Pathways in cancer	−13.5
	hsa05145	Toxoplasmosis	−13.2

MCODE3	hsa04110	Cell cycle	−4.5
	hsa04218	Cellular senescence	−4.2
	hsa04115	p53 signaling pathway	−3.1

**Table 3:** Core ingredients of *Tripterygium wilfordii*.

Molecule ID	Compound name	Degree	Betweenness centrality	Closeness centrality
MOL000422	kaempferol	98	0.77999059	0.59245283
MOL003187	Triptolide	44	0.35251619	0.41424802
MOL005828	Nobiletin	18	0.08668321	0.36596737
MOL003192	Triptonide	14	0.08281171	0.35440181
MOL000358	Beta-sitosterol	13	0.03563423	0.36091954
MOL000449	Stigmasterol	9	0.04243931	0.32640333

**Table 4:** Core target of *Tripterygium wilfordii*.

Target	Degree	Betweenness centrality	Closeness centrality
CASP3	8	0.09695571	0.50159744
PARP1	5	0.04744058	0.48307692
BCL2	5	0.04744058	0.48307692
BAX	4	0.01657181	0.39546599
TNF	4	0.03692728	0.45772595

**Table 5:** Results of molecular docking.

Chemical composition	Target	PDB ID	Molecule affinity	Active pocket site
Kaempferol	CASP3	ICP3	−8.46	G122, M61, F128, C163, A162, S205, Q161, R64, S120, G122
	PARP1	IUKO	−7.52	L877, Y896, I895, R878, G894, E763, N767, R865, H909, R878, G894, Y896

Triptolide	CASP3	ICP3	−7.67	W206, Y204, C163, A162, R207, C163, G122
	PARP1	IUKO	−8.35	Y896, Y889, I895, Y907, P881, A880, R878, D770, H862, Y896

**Table 6:** The kinetic affinities between active components and target proteins.

Chemical composition	Target	Ka (1/M ∗ S)	Kd (1/S)	KD (M)
Kaempferol	CASP3	9.50*e*2	7.27*e* − 3	7.65*e* − 6
Triptolide	PARP1	2.39*e*2	9.99*e* − 3	4.18*e* − 5

Ka: association constant; Kd: dissociation constant; KD: equilibrium dissociation constant.

## Data Availability

The data that support the findings of this study are available from the corresponding author upon reasonable request.
